# Epilepsy in Tibetan-Inhabited Plateau Regions of Western China: A Narrative Review of Epidemiology, Uncertainty About Chronic High-Altitude Effects, and Evidence-Informed Healthcare Priorities

**DOI:** 10.3390/healthcare14203511

**Published:** 2026-10-19

**Authors:** Yulong Li, Yixiu Lin, Lei Chen

**Affiliations:** 1 Department of Neurology, West China Hospital, Sichuan University, Chengdu 610000, China; 2 Engineering Research Center of Brain-Machine Interactive Neuromodulation Sichuan Provincial, Chengdu 610000, China; 3 Laboratory of Neuro-diseases and Multimorbidity, West China Hospital, Sichuan University, Chengdu 610000, China; 4 Sichuan Provincial Model Worker and Craftsman Innovation Studio, Chengdu 610000, China; 5 Department of High Altitude Medicine, Center for High Altitude Medicine, West China Hospital, Sichuan University, Chengdu 610000, China

**Keywords:** epilepsy, Tibetan-inhabited plateau regions, high altitude, healthcare access, effective coverage, healthcare delivery, treatment gap, rural health, health services, disease prevention

## Abstract

**Highlights:**

**What are the main findings?**

- Community surveys reported prevalence estimates of 2.5–23.5 per 1000, but methods and case definitions differed; no resident-population study identified in this review reported all four review-defined features used here to assess claims about chronic altitude exposure.
- Regional evidence documents selected potentially preventable causes, gaps in treatment quality and continuity, and uneven diagnostic and workforce capacity. In one assessed subgroup, 35.4% screened positive for depressive symptoms and 52.0% for anxiety symptoms.

**What are the implications of the main findings?**

- Healthcare priorities can be evaluated prospectively even while the role of chronic altitude exposure remains uncertain.
- The four review-defined appraisal features provide a transparent framework for examining claims about altitude-related health risks in remote settings.

**Abstract:**

Background/Objectives: In Tibetan-inhabited plateau regions of western China, remoteness, uneven access to care, and preventable causes complicate epilepsy care. We assessed whether regional evidence supports an independent effect of chronic altitude exposure on epilepsy risk and identified healthcare priorities. Methods: We conducted a targeted narrative review of English-language journal articles published through 12 June 2026 (PubMed/MEDLINE, Web of Science Core Collection, citation tracking). Altitude-related evidence was evaluated against four prespecified, review-defined features; core sources underwent structured qualitative appraisal. Results: Three community surveys with differing case definitions and ascertainment reported prevalence estimates of 2.5–23.5 per 1000; the most recent (Daofu County, 2025) reported 4.4 per 1000 (95% confidence interval 3.7–5.1). No population-based incidence study of long-term residents was identified among the retrieved publications, and no identified resident-population study reported all four features. Regional studies documented parasitic, traumatic, and vascular etiologies, historical treatment gaps of 97–100%, and uneven diagnostic and workforce capacity. Among 127 Daofu participants assessed in detail, 33.1% were classified as receiving nonstandard treatment and 14.2% as untreated; 35.4% and 52.0% screened positive for depressive and anxiety symptoms. Conclusions: Current evidence neither establishes nor excludes an independent effect of chronic altitude exposure. It supports prospective evaluation of prevention, treatment continuity, diagnostic and referral services, workforce development, integrated mental health care, and surveillance.

## 1. Introduction

Epilepsy imposes substantial health and societal burdens, particularly where acquired causes and gaps in treatment persist [1,2,3]. China has expanded community-based epilepsy management and specialized services over the past two decades [4]. However, access to diagnosis, treatment, and continuing care may remain uneven in remote areas, where workforce distribution and retention can constrain service delivery [5]. In Tibetan-inhabited plateau regions of western China, altitude exposure, remoteness, and healthcare access vary together. These overlapping conditions make it difficult to separate biological effects of altitude from social, diagnostic, and health service influences.

Published surveys and modeled analyses have reported substantially different estimates of epilepsy prevalence in these regions [6,7,8,9]. Geographic and environmental explanations have been proposed for regional variation [6]. However, residence in a plateau region does not establish an individual’s chronic altitude exposure. For descriptive consistency, this review uses high altitude to denote elevations of at least 2500 m when source data permit; this threshold is descriptive and does not imply a biological cutoff for epilepsy risk [10]. Epilepsy is distinguished from an isolated seizure and from an acute symptomatic seizure occurring in close temporal association with an acute insult [11,12].

Healthcare access and effective coverage are also distinct. Access reflects whether people can obtain and use services when needed. Effective coverage reflects receipt of care that is adequate to improve health outcomes, not simply contact with services [13,14,15]. These distinctions are important when interpreting regional variation in recorded prevalence, treatment, and outcomes.

This review addresses three questions: (1) what available studies show about epilepsy prevalence, etiologies, treatment, and outcomes in these regions; (2) whether available evidence supports an independent effect of chronic altitude exposure on epilepsy among long-term residents; and (3) which healthcare and public health priorities warrant evaluation despite this uncertainty. By separating unresolved altitude attribution from more directly supported healthcare needs, the review identifies priorities that can be evaluated without assuming a causal effect of altitude. Four explicitly defined features guide our assessment of evidence for a chronic-altitude effect.

## 2. Methods

We conducted a narrative review of peer-reviewed evidence relevant to epilepsy in Tibetan-inhabited plateau regions of western China. A narrative approach was appropriate because the evidence spans population surveys, clinical cohorts, modeled estimates, biological studies, and health service research, with substantial differences in populations, exposures, outcomes, and study purposes. Throughout this review, “Tibetan-inhabited plateau regions” is used as a sociogeographic descriptor. We classified studies as directly relevant when participants were sampled from the Tibet Autonomous Region or from Tibetan autonomous prefectures or counties in western China. Studies from neighboring areas or mixed populations were classified as indirect evidence unless results for the target region were reported separately; examples include a physician survey combining Lhasa and Xichang, a rural management cohort from counties outside the target plateau setting, and a military cohort outside the target region. We treated administrative location, ethnicity, place of residence, and individual altitude exposure as distinct concepts.

Targeted searches of PubMed/MEDLINE and Web of Science Core Collection were conducted on 12 June 2026. The searches combined epilepsy- and seizure-related terms with terms describing the Tibetan-inhabited plateau regions or altitude exposure; the database-specific strategies and record flow are reported in Supplementary Table S1. Backward and forward citation tracking supplemented the database searches. Eligible sources were peer-reviewed journal articles published in English through 12 June 2026. Chinese-language publications and non-journal reports were outside the scope of this review. Statements that no study of a particular design was identified are limited to the literature retrieved for this review.

We selected sources that informed epilepsy prevalence or distribution; potentially preventable or modifiable etiologies; status epilepticus; treatment continuity or effective coverage; mortality; diagnostic or workforce capacity; mental health comorbidity; biological plausibility; or seizures after recent or subacute altitude exposure. When sources addressed similar questions, we emphasized those with explicit case definitions, defined denominators, and measures of uncertainty. For core sources, we documented the setting and study period, population and residency criteria, outcome definitions and ascertainment, sample size, major estimates, relevance to chronic altitude exposure, and principal limitations.

We assessed evidence for an effect of chronic altitude exposure using four review-defined features (Table 1): temporally appropriate measurement of chronic altitude exposure; comparable epilepsy ascertainment across populations; an informative contrast in altitude exposure; and appropriate handling of major social, etiologic, diagnostic, and health system factors. The four domains were specified before appraisal, drawing on epidemiologic principles of exposure measurement, outcome ascertainment, exposure contrast, and confounding, as well as established standards for epilepsy epidemiology [16]. Their wording was refined during manuscript preparation, but the domains remained unchanged throughout the appraisal. We synthesized the findings narratively because populations, outcomes, exposure measures, and study objectives differed across sources. We did not pool community prevalence estimates because differences in case definitions, sampling, study periods, and diagnostic ascertainment made it implausible that the surveys estimated a common underlying prevalence. We report confidence or uncertainty intervals when available in the original publications and did not calculate additional intervals.

We conducted a structured qualitative appraisal of the ten sources in Table 2 and the two regional studies of parasitic disease described in Section 3.2 (Supplementary Table S2). For each source, we evaluated sampling and selection, outcome ascertainment, exposure or setting characterization, analysis and contextual factors, precision, and applicability. We also assessed sources relevant to altitude exposure against the four features in Table 1. We assigned no summary scores. The appraisal domains are not a validated risk-of-bias instrument. Yulong Li and Yixiu Lin conducted the appraisal jointly through discussion; they did not perform independent duplicate appraisals.

## 3. Results

Table 2 summarizes the main evidence and its limitations. Supplementary Table S2 presents the structured appraisal.

### 3.1. Population Epidemiology and Limits of Inference About Chronic Altitude

Three community surveys reported substantially different prevalence estimates among residents. A 2006 household survey of 14,822 rural residents reported a lifetime prevalence of convulsive epilepsy of 2.5 per 1000 (95% CI 1.8–3.4) and an active prevalence of 2.4 per 1000 (95% CI 1.7–3.3) [7]. A 2008 household survey of 7669 residents across 45 villages in two Zayul County towns reported a lifetime prevalence of convulsive epilepsy of 23.5 per 1000 (95% CI 20.1–26.9) and an active prevalence of 22.8 per 1000 (95% CI 19.5–26.1) [8]. In Daofu County, a 2025 survey of 37,390 permanent residents identified 164 cases using the 2014 International League Against Epilepsy definition. The crude prevalence was 4.4 per 1000 (95% CI 3.7–5.1) [9].

The two earlier surveys assessed convulsive epilepsy without routinely using electroencephalography (EEG) or neuroimaging to ascertain cases. In the Zayul survey, 25 of 180 cases (13.9%) were classified as alcohol-related [8]. Some alcohol-related events may have been acute symptomatic seizures, which alone do not establish a diagnosis of epilepsy [11,12]. This distinction adds uncertainty to comparisons across surveys. Among cases identified in Daofu, 82.9% reported prior computed tomography (CT) or magnetic resonance imaging (MRI), and 78.0% reported prior EEG [9]. The surveys differed in location, study period, sampling, case definition, and ascertainment. Their prevalence estimates are therefore not directly comparable. No population-based incidence study of long-term residents was identified through this review.

Participant-level residential altitude was not reported in any of the three community surveys [7,8,9]. These data cannot support an individual-level altitude–response analysis. Residence in a plateau administrative region is not a direct measure of lifetime altitude exposure.

A national synthesis reported Tibet-specific estimates ranging from 2.42 to 23.47 per 1000. The largest contributing survey reported the lowest estimate, whereas the smallest reported the highest. The high-prevalence corridor described in that synthesis was not defined by measured altitude and lay well below plateau elevations [6]. This synthesis included the two earlier surveys discussed above [7,8] and does not provide independent replication of their findings. International evidence also shows substantial heterogeneity in prevalence estimates according to rurality, study size, case definition, and ascertainment [16,17,18]. GBD 2019 modeled an age-standardized epilepsy prevalence for Tibet of 438.85 per 100,000 (95% uncertainty interval 115.95–740.52), compared with 219.69 per 100,000 nationally [19]. These GBD estimates refer to the modeled idiopathic epilepsy cause category rather than to epilepsy of all etiologies [1,19]. This modeled, province-level age-standardized estimate measures a different quantity from the crude community survey estimates; direct numerical comparison is inappropriate. The wide uncertainty interval indicates substantial imprecision.

Evidence from recent or subacute ascent concerns a different exposure and outcome. A prospective military cohort outside the target region followed 39,213 healthy adult men who had ascended from low altitude and stayed at elevations of at least 2500 m for more than 30 days. During 39,848.6 person-years, 41 new-onset seizures occurred, corresponding to 102.9 per 100,000 person-years (95% CI 75.8–139.7); 12 were associated with cerebral venous thrombosis [20]. Exposure was recorded in broad altitude categories. There was no concurrent low-altitude comparison group, and only two events occurred above 5800 m. The outcome was new-onset seizure, not incident epilepsy. The study therefore does not estimate epilepsy incidence among acclimatized long-term residents.

Hypobaric hypoxia has been proposed to modify seizure susceptibility through oxidative, inflammatory, vascular, and neuronal pathways [21,22]. However, this evidence is experimental or indirect and cannot establish whether chronic altitude exposure changes epilepsy risk among long-term residents. Physiologic and genetic adaptation among Tibetan highlanders further limits extrapolation from recent ascent to lifelong residence [23]. No study of long-term residents identified in this review reported all four review-defined features used here to evaluate causal claims about chronic altitude exposure (Table 1; Supplementary Table S2).

### 3.2. Potentially Preventable or Modifiable Etiologies

In the Daofu community survey, investigators attributed epilepsy to intracranial parasitic infection in 16.5% of identified cases, traumatic brain injury in 11.0%, and stroke in 7.3%; the cause was classified as unknown in 32.3% [9]. A retrospective comparison included 165 inpatients with epilepsy in Ganzi and 165 age- and sex-matched inpatients in Chengdu. Among patients in Ganzi, reported etiologies included head trauma in 20.6%, stroke in 10.9%, neurocysticercosis in 7.9%, cerebral echinococcosis in 6.7%, and tuberculous infection in 5.5%. Infectious etiologies were more frequent in Ganzi than in Chengdu (24.8% vs. 7.3%; unadjusted odds ratio 4.22, 95% CI 2.12–8.37) [24]. The inpatient groups were matched on age and sex, but the reported odds ratio was not adjusted using a multivariable model. The cohorts differed substantially in residence, education, occupation, ethnic composition, and diagnostic practice. The difference in infectious etiologies cannot be attributed to altitude, and the inpatient proportions should not be interpreted as population estimates.

Two regional studies provide further context on parasitic exposures and associated neurologic disease. In a survey of 661 Tibetan volunteers in one township of Yajiang County, Ganzi, 56 participants (8.5%) were identified as having symptoms suggestive of late-onset epilepsy. Among tested participants classified as having epilepsy, 9 of 55 (16.4%) were seropositive for *Taenia solium* cysticercosis antibodies, compared with 11 of 445 (2.5%) participants without epilepsy [25]. The site was selected after hospital reports of cysticercosis, and epilepsy was assessed by questionnaire and clinical examination without neuroimaging. Seropositivity alone cannot establish neurocysticercosis as the cause of epilepsy. No *T. solium* carriers were identified among 21 confirmed tapeworm carriers. These data indicate local exposure to cysticercosis but cannot establish a representative epilepsy prevalence or an etiologic fraction. In a Ganzi hospital cohort of 97 patients with intracranial alveolar echinococcosis, 20 (20.6%) had epilepsy. The investigators reported an association between cortical involvement and epilepsy in multivariable logistic regression, with a wide confidence interval; 13 of the 16 patients receiving antiseizure medication had unsatisfactory seizure control [26].

Evidence of parasitic, traumatic, and vascular etiologies in community and clinical studies suggests potential prevention priorities. Neurocysticercosis is a preventable cause of acquired epilepsy in endemic settings [27]. Clinical and imaging findings are central to diagnostic criteria; serology alone is insufficient [28,29]. Studies in endemic settings also document neurocysticercosis among people with epilepsy [30]. Control of *Taenia solium* transmission depends on the local enabling environment as well as on the technical intervention itself [31]. Echinococcosis has imposed a substantial burden in parts of the Tibetan Plateau [32] and requires species- and setting-specific control [33]. Central nervous system infection can produce acute symptomatic seizures and, after a latent interval, acquired epilepsy through inflammatory and structural injury [34]. These findings support evaluating prevention strategies but do not establish the population-attributable burden of any specific cause.

### 3.3. Treatment Continuity, Effective Coverage, and Severe Outcomes

The two historical surveys reported large treatment gaps. Both defined the treatment gap by the absence of optimal, regular antiseizure medication during the week before the survey among people with active convulsive epilepsy. By this definition, the gap was 97% (34 of 35) in the 2006 survey [7]. This does not mean that 97% received no treatment of any kind. In the Zayul survey, the gap was 100%, and none of the 175 people with active epilepsy reported taking either antiseizure medication or traditional Tibetan medicine during that week [8]. In Zayul, the surveyed health facilities, including the county hospital, did not provide conventional antiseizure medication, and no local health worker had received formal training in epilepsy care [8]. Both estimates reflect historical definitions and local service conditions, not current treatment coverage. The International League Against Epilepsy (ILAE) Epidemiology Commission has highlighted variation in treatment-gap definitions and proposed separate diagnostic and therapeutic components [35]. By contrast, the Daofu study reported treatment-status categories rather than a comparable treatment-gap estimate [9].

The more recent Daofu study documents medication use and contact with specialist care, but these measures do not establish effective coverage. In Daofu, 83.5% of all 164 identified cases were using at least one antiseizure medication, and 87.8% had sought tertiary care. Among 127 participants assessed in detail, investigators classified 33.1% as receiving nonstandard treatment and 14.2% as untreated [9]. Medication receipt alone does not establish appropriate, continuous, or effective care. Measures of effective coverage should include treatment appropriateness, retention, seizure control, adverse effects, and quality of life [14,15].

In a two-center study of status epilepticus, 223 of 318 patients admitted in Tibet (70.1%) had previously diagnosed epilepsy with inadequate seizure control. Of these, 111 had never received an antiseizure medication, and 52 (23.3%) had received treatment that the investigators judged inappropriate; 40 had used traditional Tibetan medicine alone [36]. These categories reflect the investigators’ definitions and do not establish patient nonadherence or treatment preferences. No nonconvulsive status epilepticus was recorded in Tibet, and EEG and lumbar puncture were used less often [36]. Unadjusted outcome comparisons cannot establish differences in hospital performance. These findings motivate evaluation of whether continuity of epilepsy care, rescue planning, and timely referral can prevent some episodes of status epilepticus. Historical treatment-gap estimates should not be compared directly with contemporary medication use because definitions of active, untreated, and traditionally treated epilepsy differ across studies [37,38,39].

Evidence on mortality in the target population remains indirect. GBD 2019 modeled an age-standardized mortality rate for idiopathic epilepsy in Tibet of 4.26 per 100,000 (95% uncertainty interval 1.43–5.66) [19]. This model-based population mortality rate is distinct from the mortality rate among people with epilepsy. A management cohort in 10 rural counties of Sichuan recorded 44 probable cases of sudden unexpected death in epilepsy (SUDEP) during 31,347 person-years among 10,128 people with convulsive epilepsy. The reported SUDEP incidence was lower during follow-up years 6–8 than during years 1–5 (incidence rate ratio 0.4, 95% CI 0.2–0.8) [40]. The cohort was uncontrolled and not plateau-specific, so it cannot estimate plateau SUDEP. The study nevertheless demonstrates that follow-up, medication supply, and death ascertainment can be organized in a rural management program [40]. Future regional mortality surveillance could include structured ascertainment of SUDEP [41]. More generally, effective coverage should be assessed by outcomes such as seizure control rather than medication receipt alone [14,15].

### 3.4. Diagnostic and Referral Capacity, Workforce, and Mental Health

Available studies suggest variation in diagnostic and workforce capacity but are not representative of the entire region. In the Ganzi inpatient comparison, EEG had been used in 30.9% of patients, compared with 91.5% in Chengdu [24]. A 2026 convenience survey of 325 physicians attending epilepsy training found that 73.97% of the Chengdu participants were neurologists, compared with 12.85% of the investigators’ combined plateau group [42]. The investigators reported no statistically significant differences in neurologist knowledge scores between groups. However, the plateau subgroup included only 23 neurologists, and the convenience sample cannot estimate workforce density or clinical competence. The plateau group combined Lhasa and Xichang. Because Xichang lies outside this review’s target region, the combined results provide only indirect evidence for the plateau population.

China has developed a tiered network of epilepsy centers [43]. This network could support referrals and specialist consultation, but its reach, quality, and equity in plateau counties have not been evaluated. Remote review of EEG, imaging, and clinical information and locally adapted community health worker or mobile health models are potential service approaches [44,45]. Their effectiveness in the target region remains untested.

Mental health care is an important consideration in designing epilepsy services. Among the 127 participants assessed in Daofu, 45 (35.4%; 95% CI 27.1–43.8) screened positive for depressive symptoms and 66 (52.0%; 95% CI 43.3–60.7) screened positive for anxiety symptoms [9]. These symptoms were assessed using Tibetan-language translations of the Chinese Neurological Disorders Depression Inventory for Epilepsy and the Generalized Anxiety Disorder-7 scale, respectively. Psychiatric comorbidity is more common in people with epilepsy than in the general population and can add substantially to disability and impaired quality of life [46,47]. These proportions come from one subgroup and should not be generalized to the entire regional epilepsy population. They support evaluating mental health screening and referral as part of epilepsy care.

**Table 2:** Selected Evidence Informing the Review.

Study and Design	Population and Outcome	Main Findings	Principal Inferential Limitation
Zhao et al., 2008 [7]; door-to-door survey, 2006	14,822 rural residents in the Lhasa and Chamdo areas; lifetime convulsive epilepsy	Crude prevalence 2.5/1000 (95% CI 1.8–3.4); active 2.4/1000 (95% CI 1.7–3.3); 34 of 35 active cases had not received optimal, regular ASM in the preceding week	No participant-level altitude data; few cases; EEG and imaging not routinely used to ascertain epilepsy; historical survey
Zhao et al., 2010 [8]; door-to-door survey, 2008	7669 residents across 45 villages in two Zayul County towns; convulsive epilepsy	Crude lifetime prevalence 23.5/1000 (95% CI 20.1–26.9); active 22.8/1000 (95% CI 19.5–26.1); 100% treatment gap; neither antiseizure medication nor traditional Tibetan medicine reported in the preceding week	No participant-level altitude data; no EEG or neuroimaging for case ascertainment; 13.9% classified as alcohol-related
Wang et al., 2026 [9]; community survey, 2025	37,390 permanent residents in Daofu County; ILAE-defined epilepsy (164 cases); 127 assessed in detail	Crude prevalence 4.4/1000 (95% CI 3.7–5.1); parasitic 16.5%, traumatic 11.0%, stroke 7.3%, unknown 32.3% ^a^; medication use 83.5% ^a^; nonstandard treatment 33.1% and untreated 14.2% ^b^	Single county; no altitude contrast; potential underascertainment of subtle or nonconvulsive seizures
Wu et al., 2022 [6]; national synthesis	Published prevalence surveys across China; five Tibet-specific surveys	Tibet-specific estimates 2.42–23.47/1000; the largest survey produced the lowest estimate; the proposed high-prevalence belt was not altitude-defined	Ecological synthesis; heterogeneous study periods, designs, and case definitions
Liu et al., 2023 [19]; GBD 2019 modeling	Province-level estimates for China	Modeled, age-standardized prevalence of idiopathic epilepsy in Tibet 438.85/100,000 (95% UI 115.95–740.52); age-standardized mortality rate 4.26/100,000 (95% UI 1.43–5.66)	Modeled province-level estimates; sparse regional inputs; altitude not measured as an exposure; not comparable with crude survey estimates
Chen et al., 2018 [24]; retrospective inpatient comparison	165 Ganzi and 165 age- and sex-matched Chengdu inpatients with epilepsy	Infectious etiologies 24.8% vs. 7.3% (unadjusted OR 4.22, 95% CI 2.12–8.37); EEG use 30.9% vs. 91.5%	Hospital-based selection; socioeconomic and diagnostic differences; altitude not measured
Peng et al., 2025 [36]; two-center status epilepticus study, 2015–2020	318 patients in Tibet and 301 in Chengdu with status epilepticus	223 (70.1%) had inadequately controlled preexisting epilepsy; 111 had never received antiseizure medication, and 52 (23.3%) had received treatment judged inappropriate	Different diagnostic ascertainment; no nonconvulsive status epilepticus recorded; crude outcomes do not measure comparative hospital quality
Wang et al., 2021 [40]; rural management cohort	10,128 people with convulsive epilepsy; 31,347 person-years	44 probable SUDEP cases; incidence lower in years 6–8 than in the first 5 years (IRR 0.4, 95% CI 0.2–0.8)	Not specific to the target plateau region; uncontrolled; program effects cannot be inferred
Xiao et al., 2026 [42]; physician training survey	325 physicians recruited in Chengdu, Lhasa, and Xichang	Neurologists comprised 73.97% of the Chengdu group and 12.85% of the combined plateau group; no statistically significant difference in neurologist knowledge scores reported (23 in the plateau subgroup)	Convenience sample; Xichang and Lhasa grouped; cannot estimate workforce density or competence
Srinath et al., 2022 [20]; prospective military cohort	39,213 healthy adult men after ascent to at least 2500 m; 39,848.6 person-years	41 new-onset seizures; 102.9/100,000 person-years (95% CI 75.8–139.7); 12 associated with cerebral venous thrombosis	Recent ascent rather than lifelong residence; broad exposure categories; no low-altitude comparator; new-onset seizures, not incident epilepsy

Prevalence estimates from community surveys are crude; GBD estimates are modeled, age-standardized, and refer to the idiopathic epilepsy cause category. ^a^ Denominator, 164 identified cases. ^b^ Denominator, 127 participants assessed in detail; the Daofu prevalence refers to 37,390 residents. No study of a resident population identified in this review reported all four review-defined features relevant to causal interpretation of chronic altitude exposure (Table 1; Supplementary Table S2). ASM, antiseizure medication; CI, confidence interval; EEG, electroencephalography; GBD, Global Burden of Disease; ILAE, International League Against Epilepsy; IRR, incidence rate ratio; OR, odds ratio; SUDEP, sudden unexpected death in epilepsy; UI, uncertainty interval.

## 4. Discussion

Regional studies provide stronger evidence of unmet healthcare needs and potentially modifiable causes than of an independent effect of chronic altitude exposure. Community estimates of epilepsy frequency are highly heterogeneous. Available population studies identified in this review do not combine direct, temporally appropriate exposure measurement with comparable epilepsy ascertainment, an informative altitude contrast, and adequate handling of major contextual factors. Whether chronic altitude exposure independently affects epilepsy risk remains unresolved. The evidence neither establishes nor excludes such an effect. This uncertainty does not preclude identification of healthcare priorities supported by more direct regional evidence.

Altitude, remoteness, and access to care may influence observed epilepsy patterns through distinct mechanisms. Experimental and indirect evidence suggests that chronic hypobaric hypoxia could affect seizure susceptibility [21,22]. Whether this alters epilepsy risk among long-term residents remains unknown. Terrain, travel time, clinician availability, household resources, and diagnostic capacity may also affect case detection, treatment continuity, and survival. Differences in measurement further complicate interpretation. Incomplete case finding may reduce recorded prevalence without changing disease incidence. Differences in access to EEG and imaging can affect diagnostic confirmation and etiologic classification, and therefore the comparability of recorded prevalence. Prevalence also depends on incidence, remission, and mortality [16,17,18]. Regional differences therefore cannot be attributed to altitude without an explicit causal framework and comparable measurement across populations. The same distinction matters for healthcare planning because service access, diagnostic intensity, and treatment continuity are measurable domains that can be evaluated without assuming an altitude effect. Recent-ascent cohorts, experimental studies, non-plateau cohorts, and convenience samples can inform biological hypotheses or service planning. They cannot directly estimate population-level effects among long-term residents of the target region.

The causal role of a contextual factor depends on the question being asked and when that factor occurs. Remoteness, socioeconomic conditions, and occupational or household exposure to parasites may confound an altitude–epilepsy association if they are associated with both residential altitude and epilepsy risk and are not themselves consequences of altitude. If residence at higher altitude increases travel barriers and limits access to care, healthcare access may mediate effects on subsequent epilepsy outcomes. Hypoxia-related vascular or neuronal changes are other possible mediating pathways [21,22], but their contribution to epilepsy risk among long-term residents has not been established. Genetic or physiologic adaptation, duration of residence, and age at exposure may modify any effect of hypobaric hypoxia [23]. Diagnostic capacity chiefly affects measurement: EEG and imaging availability can influence case ascertainment, etiologic classification, and recorded prevalence without necessarily changing underlying epilepsy risk [16,17,18]. Lower case detection in remote settings could reduce recorded prevalence, whereas differences in etiologic exposures could produce apparent altitude-related associations; the net direction of bias cannot be determined from the available data. Future studies should specify the hypothesized causal relationships in advance rather than adjust indiscriminately for all contextual variables.

These findings illustrate a broader challenge in remote settings: environmental exposure, remoteness, diagnostic intensity, and access to continuing care may vary together. Without separate measurement of these factors, geographic differences may be incorrectly attributed to altitude alone. The four review-defined features in Table 1 make explicit the limitations of causal claims based on geographic exposure proxies.

The available evidence provides a clearer basis for setting healthcare and public health research priorities. Prevention research can prioritize locally documented acquired causes [9,24]. Evaluation of setting-specific measures to control *Taenia solium* and *Echinococcus* transmission is warranted by locally documented parasitic etiologies [9,24,25,26], the echinococcosis burden reported for parts of the Tibetan Plateau [32], and established prevention frameworks [31,33]. Injury prevention, vascular risk reduction, and stroke care are additional priorities suggested by the traumatic and vascular etiologies reported in the local community and inpatient studies [9,24]. Epilepsy care should be assessed by effective coverage, not medication receipt alone: appropriate treatment, continuity, retention, seizure control, adverse effects, and quality of life [14,15]. Diagnostic and referral services can be evaluated using interpretable EEG availability, reporting times, completion of indicated CT or MRI, access to neuroradiology review, referral completion, and the time and cost required to reach care. Remote specialist support could expand access to expertise but should complement local services and be evaluated for accountability, data governance, and equitable access [44].

**Figure 1:**
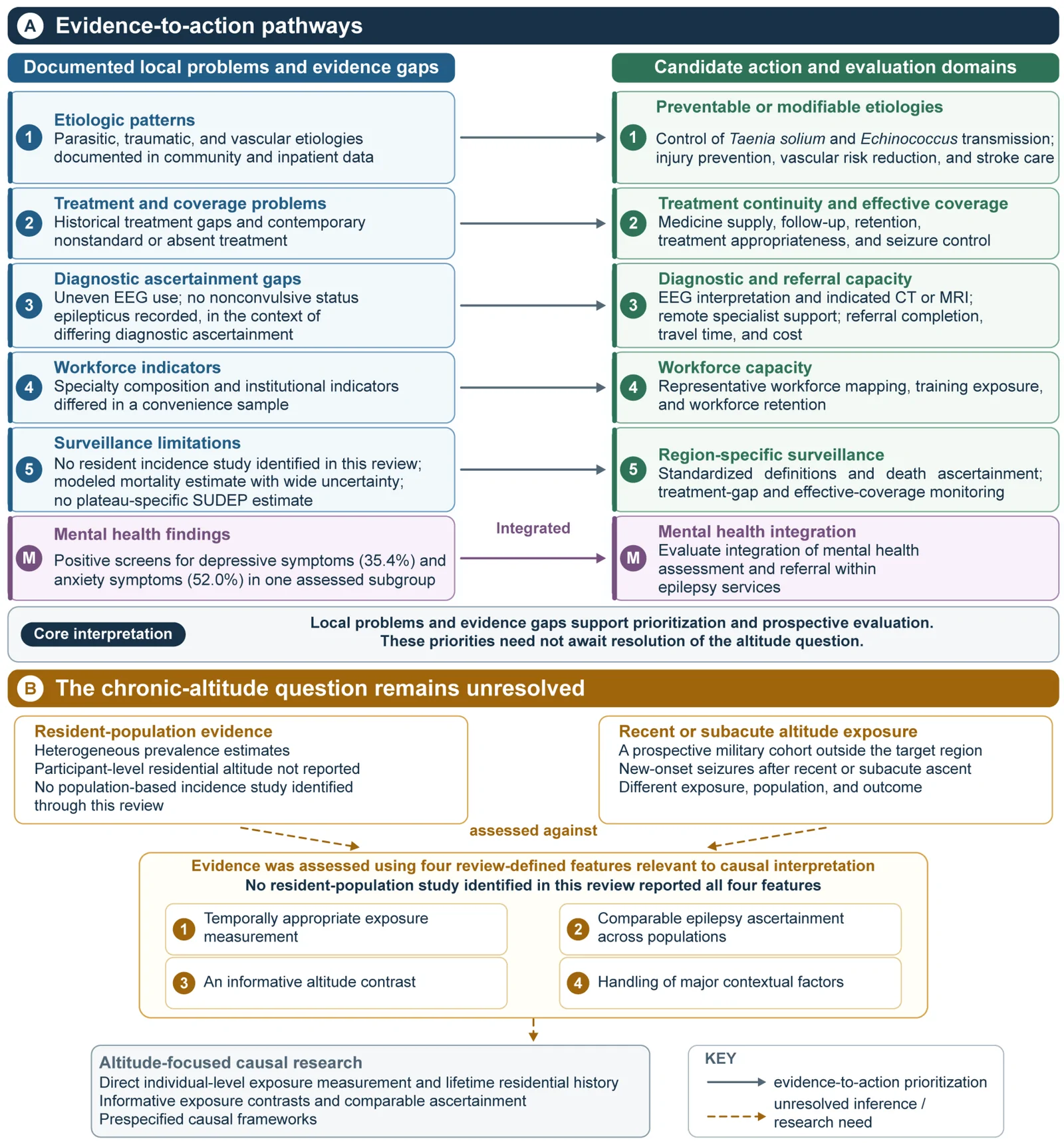
Evidence, uncertainty, and evidence-informed priorities for epilepsy care and public health in Tibetan-inhabited plateau regions of western China. (**A**) Documented local problems and evidence gaps (**left**) are linked to candidate action and evaluation domains (**right**); rows 1–5 correspond to the first five domains in Table 3, and row M addresses mental health integration. Solid arrows indicate proposed priorities for evaluation; they do not imply that an intervention is effective. (**B**) Resident-population evidence and evidence from recent or subacute ascent were appraised against four review-defined features (Table 1); these are appraisal dimensions, not a validated instrument. No resident-population study identified in this review reported all four. The bottom box identifies design elements for future studies of altitude exposure. Dashed arrows denote unresolved inference and research needs. Table 2 summarizes source studies, and Table 3 summarizes evaluation priorities. Source citations appear in the Results. CT, computed tomography; EEG, electroencephalography; MRI, magnetic resonance imaging; SUDEP, sudden unexpected death in epilepsy.

Workforce planning requires representative data on clinician distribution, training, and retention; convenience samples cannot provide these estimates [5,42]. Regional surveillance should use standardized case definitions, structured death ascertainment, and repeated measurement of prevalence, incidence when feasible, treatment gaps, and effective coverage [16,41]. Mental health assessment should be evaluated as an integral part of epilepsy services [46,47]. Figure 1 summarizes the distinction between evidence-supported healthcare priorities and the unresolved chronic-altitude question. Table 3 presents measurable domains for prospective evaluation. These domains warrant prospective evaluation; the effectiveness of specific interventions in this setting has not been established.

Evaluating healthcare interventions and testing the chronic-altitude hypothesis require different study designs. Service interventions should be evaluated using designs capable of estimating their effects. For phased implementation, stepped-wedge trials or difference-in-differences designs may be appropriate when their assumptions are satisfied. More informative evidence on chronic altitude exposure would come from multisite incidence cohorts or repeated surveys with harmonized International League Against Epilepsy case definitions and epidemiologic methods [11,12,16,48]. Such studies should measure altitude directly, record lifetime residential history and migration, and prespecify potential confounders, mediators, and effect modifiers. Informative altitude contrasts would also require comparable case ascertainment and measurement of diagnostic intensity and relevant social and health system factors.

Several limitations should be considered. The searches were targeted rather than exhaustive and were restricted to PubMed/MEDLINE, Web of Science, and English-language peer-reviewed literature; relevant studies indexed elsewhere or published in other languages may therefore have been missed. Restriction to published English-language articles introduces potential publication and language bias, particularly because some regional surveys and service reports are published only in Chinese. Statements that no qualifying study was identified should be interpreted as the yield of this review rather than proof of absence. Evidence available only in reports, policy documents, theses, websites, or conference proceedings was excluded. The included community studies used different epilepsy and treatment definitions. Clinical cohorts were subject to referral and admission selection; workforce data came from a convenience sample; and modeled estimates relied on limited regional inputs. Some studies shared investigators or institutions, and patient populations may overlap across hospital-based reports. We did not access unpublished or participant-level data. The heterogeneity of the available studies precluded a meaningful pooled prevalence estimate, and the evidence did not permit estimation of population-attributable fractions. Affordability, travel burden, stigma, language access, and cultural acceptability were not directly assessed in the included studies and warrant evaluation in the target population.

## 5. Conclusions

The studies identified in this review cannot establish whether chronic altitude exposure independently affects epilepsy incidence or prevalence among long-term residents of Tibetan-inhabited plateau regions of western China. Lack of sufficient evidence should not be interpreted as evidence of no effect. Regional studies more directly document selected infectious, traumatic, and vascular etiologies, historical treatment gaps, challenges in treatment continuity, and uneven diagnostic capacity. In one assessed community subgroup, 35.4% screened positive for depressive symptoms and 52.0% for anxiety symptoms.

Priorities for prospective evaluation include prevention of locally relevant acquired causes, treatment continuity and effective coverage, diagnostic and referral services, workforce development, mental health integration, and region-specific surveillance. These priorities can be studied without first resolving the chronic-altitude question. Studies addressing the altitude hypothesis should measure exposure directly, compare informative altitude ranges, harmonize epilepsy ascertainment, and prespecify causal models that account for remoteness, social conditions, diagnostic practices, and healthcare access. The review-defined features may also guide appraisal of future studies using geographic proxies for environmental exposure.

## Figures and Tables

**Table 1:** Review-Defined Features Used to Appraise Evidence Bearing on Chronic Altitude Exposure.

Domain	Features That Strengthen Causal Interpretation	Why It Matters in This Setting
Temporally appropriate exposure measurement	Well-characterized altitude exposure over the period relevant to epilepsy onset, preferably including individual residential history when available	Administrative region, ethnicity, or current residence does not establish lifetime altitude exposure
Comparable epilepsy ascertainment	Comparable or harmonized epilepsy case definitions and ascertainment procedures across compared populations	Differences in EEG and neuroimaging availability can affect diagnostic confirmation, etiologic classification, and the comparability of recorded prevalence
Informative altitude contrast	A substantial and well-characterized contrast in altitude exposure across compared populations	A contrast confined to a narrow or poorly characterized elevation range provides limited information about an altitude–response gradient
Handling of major contextual factors	Relevant social, etiologic, diagnostic, and health system factors specified and addressed by design or analysis	Remoteness, household resources, parasitic exposure, and diagnostic intensity may vary alongside altitude across settings

These features were defined for this review to make the basis of appraisal explicit. They are not a validated appraisal instrument and are not proposed as a reporting standard. EEG, electroencephalography.

**Table 3:** Evidence-Informed Healthcare and Public Health Priorities for Prospective Evaluation.

Domain	Locally Documented Finding	Priority for Prospective Evaluation	Suggested Evaluation Indicators
Preventable or modifiable etiologies	Parasitic, traumatic, and vascular etiologies reported in community and inpatient studies [9,24,25,26]	Evaluate setting-specific control of *Taenia solium* and *Echinococcus* transmission, together with injury prevention, vascular risk reduction, and stroke care	Incidence and proportion of newly diagnosed epilepsy with prespecified parasitic, traumatic, and vascular causes, where surveillance and etiologic ascertainment permit
Treatment continuity and effective coverage	Historical treatment gaps of 97% [7] and 100% [8]; nonstandard treatment in 33.1% and no treatment in 14.2% of assessed participants (*n* = 127) [9]	Evaluate antiseizure medication supply, follow-up, retention, and treatment appropriateness	Retention at prespecified follow-up intervals; treatment appropriateness; seizure control; adverse effects; quality of life; status epilepticus admissions among people with established epilepsy
Diagnostic and referral capacity	EEG use of 30.9% vs. 91.5% in matched inpatient cohorts; no nonconvulsive status epilepticus recorded in the Tibet cohort, in the context of differing diagnostic ascertainment [24,36]	Evaluate EEG and imaging capacity, referral pathways, and remote specialist support that complements local services	Proportion of interpretable EEG recordings; reporting time; completion of indicated CT or MRI; neuroradiology review; referral completion; travel time and cost
Workforce capacity	Physician specialties and institutional characteristics varied within a convenience sample [42]	Conduct representative workforce mapping; evaluate training, recruitment, and retention; assess locally adapted community health worker support	Clinician distribution and training exposure; retention at prespecified follow-up intervals; county-level epilepsy-specific capacity
Region-specific surveillance	No resident incidence study identified in this review; modeled mortality uncertain; no plateau-specific SUDEP estimate [19,40]	Establish representative surveillance using standardized case definitions, structured death ascertainment, and periodic assessment of the treatment gap and effective coverage	Incidence and prevalence with defined denominators; completeness of death ascertainment; SUDEP incidence; treatment gap; effective coverage
Mental health integration	Positive screens for depressive symptoms in 35.4% and anxiety symptoms in 52.0% of one assessed community subgroup (*n* = 127) [9]	Evaluate integration of mental health assessment and referral within epilepsy services	Assessment coverage; referral completion; treatment uptake; quality of life; patient-reported outcomes

These priorities warrant prospective evaluation; interventions in these domains have not been shown to be effective in the target region. Mental health is treated as an integrated component of epilepsy service delivery. CT, computed tomography; EEG, electroencephalography; MRI, magnetic resonance imaging; SUDEP, sudden unexpected death in epilepsy.

## Data Availability

No new participant-level data were collected. The search strategies and structured appraisal supporting this review are provided in Supplementary Tables S1 and S2. Study-level findings are available in the cited publications.

## Supplementary Materials

The following supporting information can be downloaded at: https://www.mdpi.com/article/10.3390/healthcare14203511/s1, Table S1: Database search strategies and record-selection summary; Table S2: Structured appraisal of core sources.
