# Supplementary material for: Epilepsy in Tibetan-Inhabited Plateau Regions of Western China: A Narrative Review of Epidemiology, Uncertainty About Chronic High-Altitude Effects, and Evidence-Informed Healthcare Priorities
Source: Healthcare (Basel). 2026 Oct 19;14(20):3511. doi: 10.3390/healthcare14203511 (PMC13704164; doi:10.3390/healthcare14203511)
Supplement: Supplementary file 1 [file healthcare-14-03511-s001.zip › Supplementary Table S1.pdf]

Supplementary Table S1. Database search strategies and record-selection summary

PubMed/MEDLINE and Web of Science Core Collection were searched on 12 June 2026. Two complementary evidence domains were addressed: (A) epilepsy in Tibetan-inhabited plateau regions and (B) seizures associated with altitude exposure. The executed Boolean search strings, database settings, and corresponding record counts are provided below.

Table S1A. Database-specific search strategies and retrieval counts

Search and database	Search field	Database filters	Records before filters	Records retrieved
A. Regional epilepsy — PubMed	Title/Abstract	English; Journal Article; publication date 1800/01/01–2026/06/12	38	33
B. Altitude exposure — PubMed	Title/Abstract	English; Journal Article; publication date 1800/01/01–2026/06/12	105	93
A. Regional epilepsy — Web of Science Core Collection	Topic (TS)	English; Article or Review Article; all years	57	50
B. Altitude exposure — Web of Science Core Collection	Topic (TS)	English; Article or Review Article; all years	124	112

The four searches yielded 288 records after application of the database filters shown above (126 from PubMed/MEDLINE and 162 from Web of Science Core Collection), before deduplication.

Exact Boolean search strings

PubMed/MEDLINE — Search A

(epilep*[Title/Abstract] OR seizure*[Title/Abstract] OR "status epilepticus"[Title/Abstract]) AND (Tibet*[Title/Abstract] OR Xizang[Title/Abstract] OR "Tibet Autonomous Region"[Title/Abstract] OR "Tibetan Plateau"[Title/Abstract] OR "Qinghai-Tibetan Plateau"[Title/Abstract] OR "Qinghai-Tibet Plateau"[Title/Abstract] OR "Qinghai-Xizang Plateau"[Title/Abstract] OR "Tibetan Autonomous Prefecture"[Title/Abstract] OR "Tibetan Autonomous County"[Title/Abstract] OR Ganzi[Title/Abstract] OR Garze[Title/Abstract] OR Daofu[Title/Abstract] OR Zayul[Title/Abstract] OR Chayu[Title/Abstract] OR Lhasa[Title/Abstract] OR Qamdo[Title/Abstract] OR Chamdo[Title/Abstract] OR Nagqu[Title/Abstract] OR Naqu[Title/Abstract] OR Shigatse[Title/Abstract] OR Xigaze[Title/Abstract]) AND english[Language] AND "journal article"[Publication Type] AND ("1800/01/01"[Date - Publication] : "2026/06/12"[Date - Publication])

PubMed/MEDLINE — Search B

(epilep*[Title/Abstract] OR seizure*[Title/Abstract]) AND ("high altitude"[Title/Abstract] OR "high-altitude"[Title/Abstract] OR hypobaric[Title/Abstract] OR "hypobaric hypoxia"[Title/Abstract] OR "altitude exposure"[Title/Abstract] OR "high-altitude exposure"[Title/Abstract] OR "recent ascent"[Title/Abstract] OR acclimati*[Title/Abstract]) AND english[Language] AND "journal article"[Publication Type] AND ("1800/01/01"[Date - Publication] : "2026/06/12"[Date - Publication])

Web of Science Core Collection — Search A

TS=((epilep* OR seizure* OR "status epilepticus") AND (Tibet* OR Xizang OR "Tibet Autonomous Region" OR "Tibetan Plateau" OR "Qinghai-Tibetan Plateau" OR "Qinghai-Tibet Plateau" OR "Qinghai-Xizang Plateau" OR "Tibetan Autonomous Prefecture" OR "Tibetan Autonomous County" OR Ganzi OR Garze OR Daofu OR Zayul OR Chayu OR Lhasa OR Qamdo OR Chamdo OR Nagqu OR Naqu OR Shigatse OR Xigaze))

Web of Science Core Collection — Search B

TS=(epilep* OR seizure*) AND TS=("high altitude" OR "high-altitude" OR hypobaric OR "hypobaric hypoxia" OR "altitude exposure" OR "high-altitude exposure" OR "recent ascent" OR acclimati*)

Table S1B. Record-selection summary

Search and selection stage	Records
Records retrieved from four database searches	288
Duplicate records removed	109
Unique records after deduplication	179
Records screened by title and abstract	179
Records corresponding to cited sources	14
Records not cited	165

Of the 179 records screened by title and abstract, 14 corresponded to sources cited in this review and 165 were not cited. The counts describe the database record flow, not the full set of sources identified through citation tracking.

Search and selection notes

The exact database-specific search expressions are shown above. Eligible sources were English-language journal articles published through 12 June 2026.

PubMed used Title/Abstract search fields and the publication-date range embedded in each search string. Web of Science used the Topic (TS) field and the language and document-type filters shown in Table S1A; the publication cutoff was applied during record eligibility assessment.

Duplicate publications were identified using DOI or PMID and bibliographic title/year information. Records were assessed by title and abstract for their relevance to the review questions and the defined geographic and evidence domains.

Backward and forward citation tracking supplemented the database searches. Sources identified exclusively through citation tracking are not included in the database record counts in Table S1B.
