# Supplementary material for: Epilepsy in Tibetan-Inhabited Plateau Regions of Western China: A Narrative Review of Epidemiology, Uncertainty About Chronic High-Altitude Effects, and Evidence-Informed Healthcare Priorities
Source: Healthcare (Basel). 2026 Oct 19;14(20):3511. doi: 10.3390/healthcare14203511 (PMC13704164; doi:10.3390/healthcare14203511)
Supplement: Supplementary file 1 [file healthcare-14-03511-s001.zip › Supplementary Table S2.pdf]

Supplementary Table S2. Structured appraisal of core sources

Twelve selected evidence sources were appraised descriptively, without summary scores. No resident-population study identified in this review reported all four review-defined features relevant to the causal interpretation of chronic altitude exposure.

Table S2A. Descriptive appraisal of core sources

The three community surveys differed in case definitions, diagnostic resources, and precision reporting. None directly measured participant-level residential altitude or included a comparable lower-altitude population.

Source (design)	Sampling and selection	Outcome ascertainment	Exposure or setting characterization	Analysis and handling of contextual factors	Precision	Applicability and use in this review
Zhao et al., 2008 [7] (door-to-door survey, 2006)	Randomized cluster-sampled door-to-door survey; 14,822 rural residents (Lhasa and Chamdo)	Questionnaire and clinical assessment of convulsive epilepsy; EEG/imaging not routinely used for ascertainment	Administrative area only; no participant-level altitude	Descriptive	37 lifetime cases; lifetime prevalence 2.5 per 1000 (95% CI 1.8–3.4); active prevalence 2.4 per 1000 (95% CI 1.7–3.3)	Direct; historical frequency and treatment gap
Zhao et al., 2010 [8] (door-to-door survey, 2008)	Door-to-door screening of 7669 residents in 1420 households across 45 villages of Shang and Xia Zayul towns	Two-stage: health-worker screening questionnaire, then face-to-face neurologist confirmation; no EEG or neuroimaging; 180 convulsive cases (with or without other seizure types), 175 active	Administrative area only; no participant-level altitude	Descriptive	Lifetime convulsive-epilepsy prevalence 23.5 per 1000 (95% CI 20.1–26.9); active prevalence 22.8 per 1000 (95% CI 19.5–26.1)	Direct; historical frequency, treatment gap and service capacity
Wang et al., 2026 [9] (community survey, 2025)	37,390 permanent residents of Daofu County	ILAE 2014 definition; 164 cases; detailed assessment in 127; subtle/nonconvulsive seizures may be missed	Single county; no participant-level altitude	Descriptive; subgroup with different denominator	95% CI reported for prevalence	Direct; frequency, etiology, treatment status and mental health
Wu et al., 2022 [6] (national synthesis)	Published prevalence surveys across China; five Tibet-specific	Heterogeneous case definitions and periods	Ecological; regional pattern not altitude-defined	Sample-size-weighted survey aggregation; ecological factors examined, but no participant-level altitude or confounder analysis	Tibet-specific aggregate prevalence 5.32 per 1000 (403/75,693)	Indirect (ecological); includes earlier regional surveys and is not an independent replication
Liu et al., 2023 [19] (GBD 2019 modeling)	Province-level modeled estimates	Modeled idiopathic epilepsy cause category; not ascertained directly	Province; altitude not modeled as an exposure	Age-standardized	Wide uncertainty intervals; sparse regional inputs	Indirect (modeled); burden context only
Chen et al., 2018 [24] (retrospective inpatient comparison)	165 Ganzi and 165 age- and sex-matched Chengdu inpatients	Hospital diagnosis; EEG used in 30.9% vs. 91.5%	Hospital setting; no altitude measurement	Crude comparison; age/sex matched; groups differed in residence, education, occupation, ethnicity and diagnostic practice	95% CI for infectious-etiology OR	Direct for etiology spectrum among inpatients; not population proportions
Peng et al., 2025 [36] (two-center status epilepticus study, 2015–2020)	318 inpatients in Tibet and 301 in Chengdu	Differences in diagnostic ascertainment; no nonconvulsive status epilepticus recorded in Tibet	Hospital setting; no altitude measurement	Crude comparisons	Counts and proportions	Direct for prior treatment status; outcomes not comparable between centers
Wang et al., 2021 [40] (rural management cohort)	10,128 people with convulsive epilepsy in 10 rural Sichuan counties	Probable SUDEP by structured death ascertainment	Non-plateau setting	Uncontrolled comparison across follow-up periods	IRR with 95% CI	Indirect; feasibility of follow-up and death ascertainment
Xiao et al., 2026 [42] (physician survey)	325 physicians attending training (convenience sample)	Questionnaire on epilepsy knowledge	Lhasa combined with Xichang (outside target setting)	Group comparisons; 23 neurologists in plateau subgroup	Small subgroup	Indirect; workforce indicators only
Srinath et al., 2022 [20] (prospective military cohort)	39,213 healthy men after ascent to ≥2500 m for >30 days	New-onset seizure, not epilepsy	Broad altitude categories; recent or subacute ascent; outside target region	No low-altitude comparison group	Incidence with 95% CI; 41 events, 2 above 5800 m	Indirect; recent-ascent exposure, not lifelong residence
Li T et al., 2006 [25] (community survey, 2005)	661 self-selected volunteers (60.8% of one Yajiang township); location selected after hospital cysticercosis reports	Late-onset epilepsy identified by questionnaire/clinical examination; serology for cysticercosis; no neuroimaging	County average altitude only	Serology–epilepsy association adjusted for age and sex	OR with 95% CI	Direct for local parasitic exposure; not a population prevalence or etiologic fraction

Source (design)	Sampling and selection	Outcome ascertainment	Exposure or setting characterization	Analysis and handling of contextual factors	Precision	Applicability and use in this review
Li S et al., 2021 [26] (retrospective hospital cohort, 2015–2019)	97 patients with intracranial alveolar echinococcosis, Ganzi prefectural hospital	ILAE 2014 definition; EEG not routinely used	Hospital setting; no altitude measurement	Logistic regression for lesion location; same institution/investigators as [24]	Wide CI for main association	Direct for echinococcosis-related epilepsy; clinical cohort, not population estimate

Table S2B. Review-defined features relevant to chronic altitude exposure

Ten of the twelve core sources were assessed against the four features in Table 1; none reported all four, and no source measured participant-level altitude over the period relevant to epilepsy onset. The two remaining sources, which did not address altitude-related inference, are listed as not applicable.

Source	1. Temporally appropriate exposure measurement	2. Comparable epilepsy ascertainment	3. Informative altitude contrast	4. Handling of major contextual factors	All four reported
Zhao et al., 2008 [7]	No: administrative area only	No: only one population; convulsive outcome and limited EEG/imaging	No: no contrast reported	No	No
Zhao et al., 2010 [8]	No: administrative area only	No: only one population; convulsive outcome, without EEG or imaging	No: no contrast reported	No	No
Wang et al., 2026 [9]	No: administrative area only	No: single population	No: single county	No	No
Wu et al., 2022 [6]	No: ecological, survey-level	No: definitions and periods differ across surveys	No: regional pattern not defined by measured altitude	No	No
Liu et al., 2023 [19]	No: province-level	No: modeled outcome estimates, not uniform individual ascertainment across measured altitude groups	No: altitude not modeled as an exposure	No: age-standardized estimates reported, but major contextual factors relevant to the altitude–epilepsy association were not addressed	No
Chen et al., 2018 [24]	No: hospital location only	No: EEG use 30.9% vs. 91.5%	Partly: plateau versus Chengdu setting, altitude not measured	No: unadjusted; groups differed in residence, education, occupation, ethnicity and diagnostic practice	No
Peng et al., 2025 [36]	No: hospital location only	No: different diagnostic ascertainment	Partly: Tibet versus Chengdu setting, altitude not measured	No: crude comparisons	No
Li T et al., 2006 [25]	No: county average altitude only	No: single population; questionnaire-based, without imaging	No: no contrast	No: adjustment addressed the serology–epilepsy association, not altitude	No
Li S et al., 2021 [26]	No: hospital location only	No: single cohort	No: no contrast	No: adjustment addressed lesion site, not altitude	No
Srinath et al., 2022 [20] (recent ascent; not a resident population)	Partly: broad altitude categories during recent or subacute exposure	No: no comparison population	Partly: categories within an exposed cohort; no low-altitude comparison; two events above 5800 m	No	No
Wang et al., 2021 [40] (rural management cohort)	Not applicable: the study followed deaths in people with convulsive epilepsy in rural Sichuan counties outside the target plateau setting and did not address epilepsy frequency or outcomes in relation to altitude exposure.				
Xiao et al., 2026 [42] (physician survey)	Not applicable: the study surveyed physicians' epilepsy knowledge and workforce characteristics and did not address epilepsy frequency or outcomes in relation to altitude exposure.				

Notes

1. Core sources are the ten studies in Table 2 and the two regional studies of parasitic disease described in Section 3.2. Background, guideline, mechanistic and methodological references were not appraised.
2. Appraisal is descriptive and based on the published reports. No summary score or overall quality grade was assigned, and the domains are not a validated risk-of-bias instrument.
3. Zhao 2008 [7] used randomized cluster sampling and reported 95% CIs for lifetime and active prevalence; Zhao 2010 [8] screened residents of 45 villages in two towns and reported 95% CIs for lifetime and active prevalence. All intervals are as reported by the original authors; none were calculated for this review.
4. Applicability: direct when the sampled population was reported from the Tibet Autonomous Region or a Tibetan autonomous prefecture or county in western China; indirect when the setting lay outside this region, combined target and non-target populations without separate results, or the estimate was modeled or ecological.
5. Table S2B ratings: Yes, the feature was reported; Partly, it was reported incompletely or only at group or setting level; No, it was not reported or the design did not allow it. A single-population study was rated No for comparable ascertainment and informative contrast, because neither can be assessed without a comparison. Not applicable: the study did not address epilepsy frequency or outcomes in relation to altitude exposure, so the features could not be rated.
6. Yulong Li and Yixiu Lin appraised the sources collaboratively through discussion; no independent duplicate appraisal.
7. Abbreviations: CI, confidence interval; EEG, electroencephalography; GBD, Global Burden of Disease; ILAE, International League Against Epilepsy; IRR, incidence rate ratio; OR, odds ratio; SUDEP, sudden unexpected death in epilepsy.
